# Impact of gastric and bowel surgery on gastrointestinal drug delivery

**DOI:** 10.1007/s13346-022-01179-6

**Published:** 2022-05-18

**Authors:** Susan Hua, Ephraem C. Lye

**Affiliations:** 1 Therapeutic Targeting Research Group, School of Biomedical Sciences and Pharmacy, University of Newcastle, Callaghan, NSW Australia; 2 Hunter Medical Research Institute, New Lambton Heights, NSW Australia; 3 Faculty of Medicine, University of Queensland, Brisbane, QLD Australia; 4 Department of General Surgery, Hervey Bay Hospital, Hervey Bay, QLD Australia

**Keywords:** Gastrointestinal, Surgery, Gastric, Small and large bowel, Drug delivery, Nanomedicine

## Abstract

General surgical procedures on the gastrointestinal tract are commonly performed worldwide. Surgical resections of the stomach, small intestine, or large intestine can have a significant impact on the anatomy and physiological environment of the gastrointestinal tract. These physiological changes can affect the effectiveness of orally administered formulations and drug absorption and, therefore, should be considered in rational drug formulation design for specific pathological conditions that are commonly associated with surgical intervention. For optimal drug delivery, it is important to understand how different surgical procedures affect the short-term and long-term functionality of the gastrointestinal tract. The significance of the surgical intervention is dependent on factors such as the specific region of resection, the degree of the resection, the adaptive and absorptive capacity of the remaining tissue, and the nature of the underlying disease. This review will focus on the common pathological conditions affecting the gastric and bowel regions that may require surgical intervention and the physiological impact of the surgery on gastrointestinal drug delivery. The pharmaceutical considerations for conventional and novel oral drug delivery approaches that may be impacted by general surgical procedures of the gastrointestinal tract will also be addressed.

**Graphical abstract:**

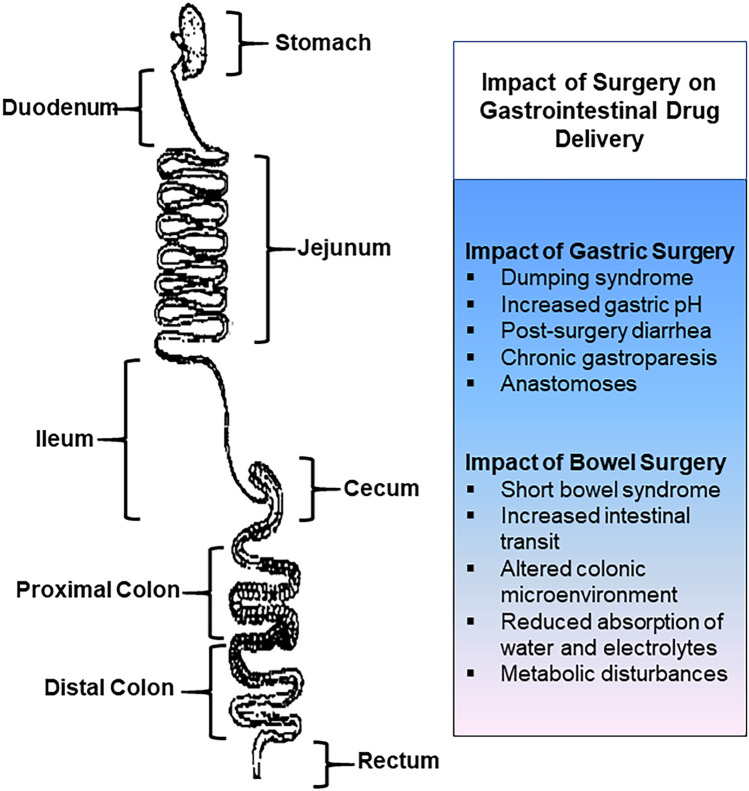

## Introduction

General surgical procedures on the gastrointestinal tract are commonly performed worldwide. These procedures can have significant implications on the anatomy and physiological environment of the gastrointestinal tract. From a pharmaceutical perspective, surgical interventions on the stomach and bowel can affect the way the body processes drugs and oral dosage forms [1–3]. The oral route is by far the most common and preferred route for drug administration by patients, due to its advantages such as non-invasiveness, ease of use, and convenience for self-administration [4, 5]. Oral formulations can be designed to enhance drug delivery to specific regions in the upper or lower gastrointestinal tract and can be used for systemic drug delivery or for treating local gastrointestinal diseases [3].

Normally, the stomach is responsible for the digestion of ingested food owing to the acid and enzymes (e.g., peptidases) in this region of the gastrointestinal tract [3]. It also acts as a temporary reservoir to control the rate of delivery of food and pharmaceuticals to the duodenum [3]. Although most drugs are minimally absorbed in the stomach, gastric resections and bariatric surgeries can affect gastric emptying and transit time [3]. The small intestine is where digestion is completed with enzymes from the liver and the pancreas. It is also the major site for the absorption of nutrients and drugs [6]. The large intestine is the final major part of the gastrointestinal tract. It is responsible for absorbing any remaining nutrients and water back into the system to maintain homeostasis, and the remaining waste product is then sent to the rectum for discharge from the body [6]. The colorectal region of the gastrointestinal tract is also a site for drug delivery and absorption [3, 7].

For optimal drug delivery, it is important to understand how different surgical procedures affect the short-term and long-term functionality of the gastrointestinal tract. This review will focus on the common pathological conditions affecting the stomach and bowel that may require surgical intervention and the physiological impact of the surgery on gastrointestinal drug delivery. The pharmaceutical considerations influencing drug delivery, including conventional and novel drug delivery approaches, will also be addressed.

## Surgical interventions for common gastrointestinal conditions

Surgical resections of or parts of the stomach, small intestine, or large intestine can have a significant impact on the anatomy and physiology of the gastrointestinal tract, which may in turn influence the effectiveness of oral formulations and drug absorption. This section will focus on the common pathological conditions affecting the gastric and bowel regions that may require surgical intervention.

### Peptic ulcers

Peptic ulcers may occur in the esophagus, stomach, and duodenum. It can also occur in the jejunum after a gastrojejunostomy, or the ileum secondary to ectopic gastric mucosa in the Meckel diverticulum [8]. In general, peptic ulcers result from the interaction of acidic gastric juice with the epithelium lining the lumen of the gastrointestinal tract [9]. The corrosive action of the gastric juice can lead to an ulcerative process, resulting in pain, bleeding, perforation, and/or obstruction. Obstruction tends to occur from inflammation and scarring in regions where the lumen is the narrowest, such as the pylorus or gastroesophageal junction [8, 9].

Medical management is the mainstay for the treatment of peptic ulcers, with the use of drugs that reduce acid production and treat *Helicobacter pylori* infection [8, 10]. Surgical therapy is primarily recommended for the treatment of complications arising from peptic ulcers (e.g., bleeding, perforation, and obstruction). However, if ulcers persist despite medical treatment, surgery may be considered to attenuate gastric acid secretion [11, 12]. The surgical procedures that are used to treat peptic ulcers are dependent on their location, with the main procedures being vagotomy with or without antrectomy [11, 12]. Both procedures can be performed laparoscopically. Excision of the ulcer itself is usually inadequate due to the likelihood of recurrence [11, 12].

Truncal vagotomy involves the resection of a small segment from each vagal trunk as it enters the abdomen on the distal esophagus, thereby causing vagal denervation of the gastric musculature [11, 12]. This leads to delayed emptying of the stomach in many patients unless a drainage procedure is performed (e.g., pyloroplasty or gastrojejunostomy) [11, 12]. Selective vagotomy of certain branches of the vagal nerves can reduce the associated morbidity [13]. For example, parietal cell vagotomy can preserve antral innervation and, therefore, ensure relatively normal gastric emptying [13]. Antrectomy involves surgical removal of the distal 50% of the stomach, which contains the gastrin-producing mucosa [13]. The proximal portion of the stomach may be reanastomosed to the duodenum or to the side of the proximal jejunum (gastrojejunostomy). In patients who have undergone multiple failed surgical procedures for refractory ulcer disease, subtotal gastrectomy may be considered [11, 12]. This procedure involves resection of a larger portion of the distal stomach (approximately two-thirds to three-fourths).

### Gastric carcinoma

Gastric carcinoma is a specific sub-group of stomach cancer that encompasses a number of different subtypes [14, 15]. These cancers usually begin in the epithelial cells that line the stomach. Gastric epithelial cancers are typically adenocarcinomas. It should be noted that squamous cell tumors of the proximal stomach involve the stomach secondarily from the esophagus. For the past several decades, cancer in the cardia region of the stomach (top part of the stomach that meets the lower end of the esophagus) has become much more common [14]. Conversely, rates of cancer in the main part of the stomach (body and antrum) have been falling worldwide [14]. Extension of the cancer occurs by intramural spread, direct extraluminal growth, and lymphatic metastases. Within the stomach, proximal spread exceeds distal spread due to the pylorus acting as a partial barrier.

Patients are generally treated with chemotherapy and surgical resection [15–17]. The surgical procedure involves removal of the tumor, an adjacent uninvolved margin of the stomach (and esophagus or duodenum depending on the location), the regional lymph nodes, and any portions of affected adjacent organs [15, 16]. Total gastrectomy is required for tumors of the proximal half of the stomach and for extensive tumors (e.g., linitis plastica). The gastrointestinal tract is then reconstructed after gastrectomy to ensure alimentary continuity (e.g., esophagojejunostomy) [15]. It should be noted that there is no nutritional value in the construction of an intestinal pouch as a substitute food reservoir following a gastrectomy. This procedure can also increase the risk of immediate complications and, therefore, is not recommended [15].

### Bariatric surgery

**Fig. 1:**
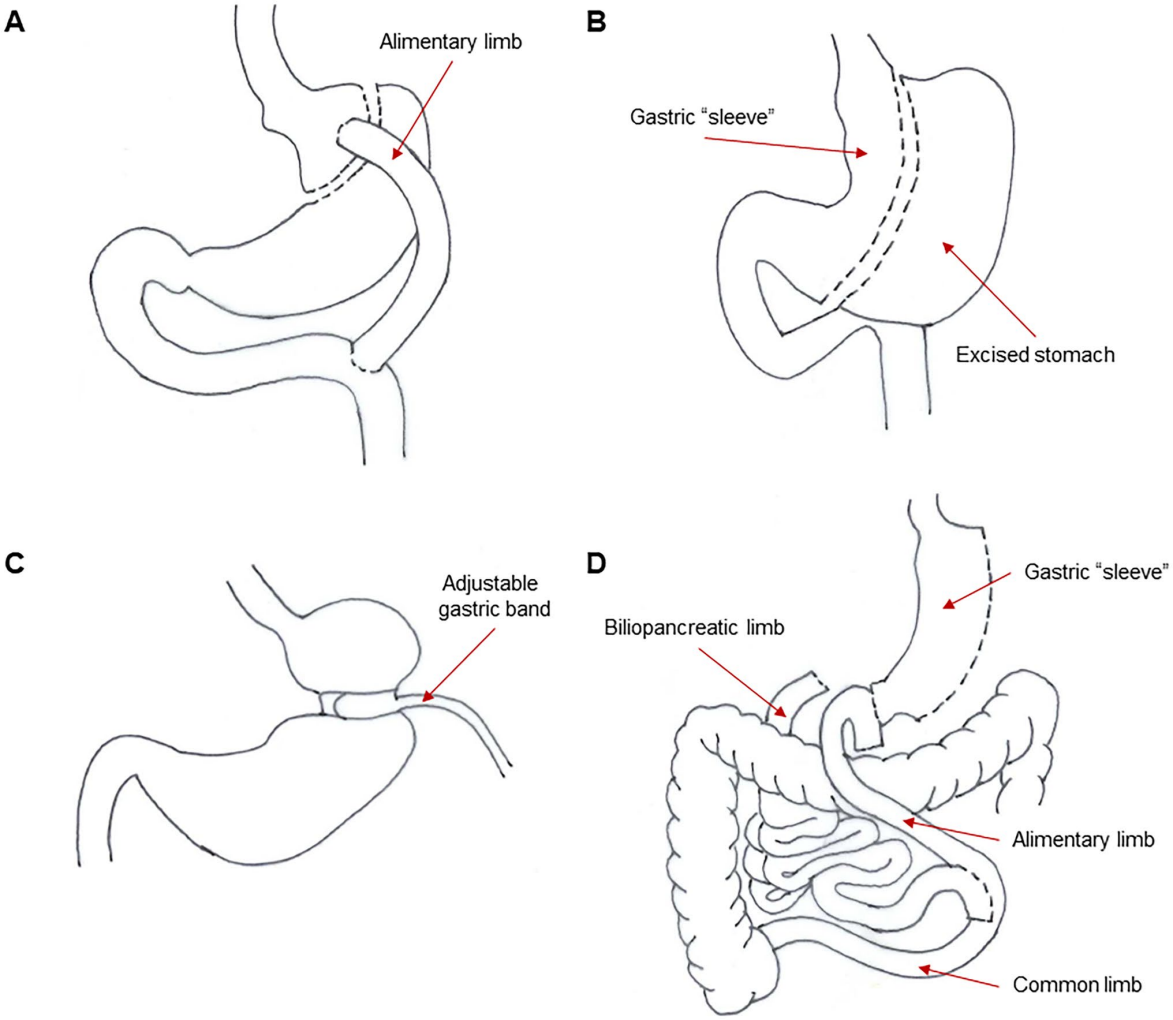
Main types of bariatric procedures. **A** Roux-en-Y gastric bypass (RYGB). **B** Sleeve gastrectomy. **C** Laparoscopic adjustable gastric banding (LAGB). **D** Biliopancreatic diversion (BPD)

Bariatric surgery is a surgical intervention to treat obesity. The general goal is to create a negative energy balance by restricting caloric intake, reducing caloric absorption, or a combination of both. Neuroendocrine changes post-surgery (e.g., gut hormones) have also been suggested to contribute to weight loss [18, 19]. The main types of bariatric procedures include gastric restriction with some malabsorption (e.g., Roux-en-Y gastric bypass (RYGB) and sleeve gastrectomy), gastric restriction with intestinal malabsorption (e.g., biliopancreatic diversion (BPD)), and gastric restriction (e.g., laparoscopic adjustable gastric banding (LAGB)) (Fig. 1) [20, 21]. These procedures are commonly performed laparoscopically, which has been reported to reduce complications and improve outcomes [22].

The RYGB procedure [20, 21] is one of the most frequently performed bariatric surgeries (Fig. 1). It involves the construction of a small 30-mL gastric pouch to which a Roux loop of jejunum is anastomosed. The small bowel is then reconstructed to form a short biliopancreatic limb for the flow of bile and pancreatic secretions, and an alimentary limb in which food from the gastric pouch travels. Both small bowel limbs then merge into a common limb that consists of the remainder of the small bowel just distal to the anastomoses. The majority of the upper gastrointestinal tract (gastric antrum, duodenum, part of the proximal jejunum) is bypassed in this configuration.

Sleeve gastrectomy [23–25] has become increasingly popular in recent years due to its effectiveness in weight loss as well as improvement in morbidity and recovery (Fig. 1). It can be performed as a single procedure in which the majority of the stomach on the greater curve side is removed (common), or as a two-stage procedure with the sleeve gastrectomy converted to RYGB or BPD after 6–12 months. BPD is similar to the RYGB procedure [20, 21]; however, it consists of a subtotal gastrectomy that reduces the gastric capacity to approximately 200- to 400-mL (Fig. 1). The relatively large gastric remnant does little to restrict food intake; therefore, the procedure primarily produces weight loss by inducing malabsorption. The small bowel is then reconstructed to form three small bowel limbs: a long biliopancreatic limb, an alimentary limb, and a common limb whereby most of the digestion and absorption occurs.

The LAGB procedure [20, 21] involves the placement of a silicone band around the upper part of the stomach (antrum), which divides the stomach into a small 20- to 30-mL proximal pouch and a larger distal stomach remnant (Fig. 1). The band can be tightened or loosened depending on the adequacy of weight loss. This procedure is relatively fast and safe; however, weight loss is significantly slower and frequently less than that achieved by other bariatric procedures. The popularity of this procedure has significantly decreased due to complications such as the band slipping out of position, gastroesophageal reflux disease (GERD), and esophageal dilations.

### Bowel obstruction

Bowel obstruction is characterized by an impairment in the normal flow of intraluminal contents. Intestinal obstruction in the small bowel is a relatively common condition that is caused by either mechanical obstruction (extrinsic or intrinsic) or paralytic ileus (neurogenic failure of peristalsis) [26, 27]. The obstruction may be restricted to the lumen; however, in more severe cases, it can also impair the blood supply leading to obstruction with strangulation and necrosis of the intestinal wall. Examples of common etiologies are intra-abdominal hernias (e.g., inguinal, femoral, and umbilical hernias), adhesions (esp. secondary to adhesions related to prior abdominal surgery), and neoplasms [26–28]. Although the exact surgical procedure will vary depending on the etiology, all small bowel loops must be examined and nonviable segments resected. If the cause of the obstruction cannot be removed (e.g., cancer infiltrating vital structures), an anastomosis bypassing the obstruction or a stoma may be considered [26–28].

With regard to obstructions in the large intestine, common causes include mechanical problems (e.g., volvulus, intussusception, and incarcerated hernia), pathology of the bowel wall (e.g., strictures and malignancy), and intraluminal factors (e.g., fecal or foreign body impaction) [28–30]. The competency of the ileocecal valve is an important factor in the clinical course of this condition. If the ileocecal valve does not allow reflux to occur (closed loop obstruction), this can lead to a rapid increase in intraluminal pressure. This pressure can lead to impaired capillary circulation, mucosal ischemia, bacterial translocation with systemic toxicity, gangrene, and perforation [29, 30]. Surgical intervention may be required for the removal of the obstructing lesion (if possible), resection of necrotic bowel tissue, and decompression of the obstructed segment. Options include resection with primary anastomosis, resection with diversion (e.g., ileostomy or colostomy), diversion alone, and endoscopic stent placement [29, 30].

### Inflammatory bowel disease

Inflammatory bowel disease (IBD) is an umbrella term for a group of chronic gastrointestinal diseases which include ulcerative colitis (UC) and Crohn’s disease (CD). The IBD subtypes are characterized by cycles of relapsing and remitting mucosal inflammation and share many clinical features [31]. Crohn’s inflammation is generally discontinuous and can affect any region of the gastrointestinal tract (terminal ileum and the colon are commonly affected), whereas the inflammation in UC is continuous and generally confined to the colon, with pancolitis (inflammation of the entire colon) occurring in some cases [31]. In severe disease, the full thickness of the intestinal wall can be inflamed, thereby increasing the risk of ulcerations, fibrotic scarring, dilation, and perforation [31]. The underlying etiology of IBD is thought to be due to a combination of environmental, genetic, microbiome, and host immune response factors [31–33].

Indications for surgical intervention include emergency surgery for patients who are severely ill or develop complications during a flare (e.g., perforation, hemorrhage, obstruction, and fistula), patients who have chronic intractable disease unresponsive to medical therapy, and treatment for dysplasia or carcinoma [34, 35]. The two main surgical options for UC are proctocolectomy with ileal pouch-anal anastomosis (IPAA) to provide intestinal continuity (most common), and total proctocolectomy with a permanent end ileostomy [34, 35]. Proctocolectomy involves mobilizing and resecting the entire colon and rectum. A subtotal colectomy may be performed in patients with severe UC with toxic megacolon, or in patients who have not completed childbearing due to the risk of impairment to sexual function and fertility following pelvic dissection for proctectomy [36].

**Fig. 2:**
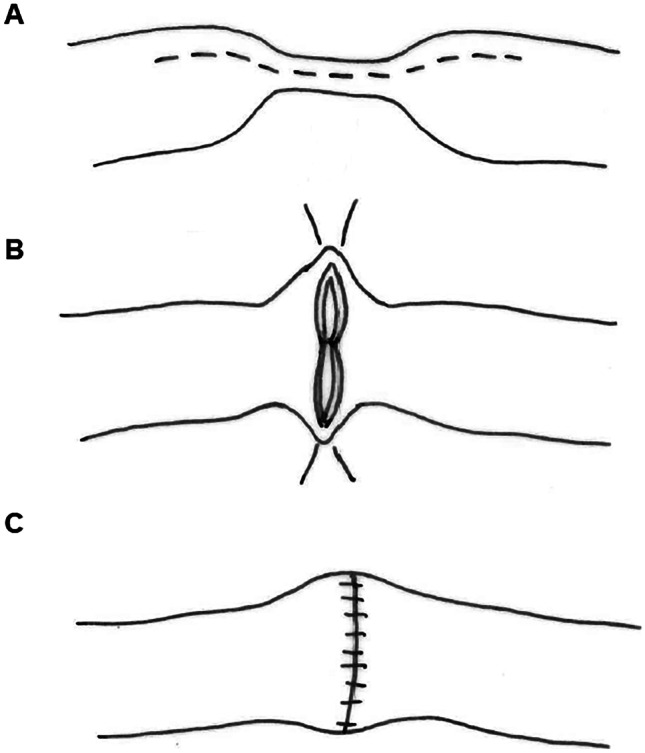
Strictureplasty. Surgical procedure performed to alleviate narrowing of the intestine due to scar tissue that has built up from chronic inflammation. Strictures are repaired by widening the narrowed area without removing any portion of the intestine. The classic Heineke–Mikulicz strictureplasty procedure involves a single longitudinal incision over the strictured area (**A**) and transverse closure of the enterotomy (**B** and **C**)

The general aim of surgical therapy for CD is to only resect the areas of severe and symptomatic disease and leave segments of bowel tissue that are mildly affected with asymptomatic disease [34]. The most common surgical procedure for CD affecting the small bowel is intestinal resection with anastomosis. The anastomosis may need to be protected with a proximal loop stoma in patients with severe scarring, sepsis, malnutrition, or recent treatment with immunosuppressants (e.g., methotrexate or infliximab) [34, 35]. Alternatively, instead of an anastomosis, the intestinal resection can be brought out to an end stoma [34, 35]. Strictureplasty may be performed in cases requiring resection of a long segment of bowel tissue, for short recurrent disease at a previous ileocolic or enteroenteric anastomosis, or for CD of the duodenum which typically manifests with stricturing disease (Fig. 2) [37, 38].

### Bowel cancer

**Fig. 3:**
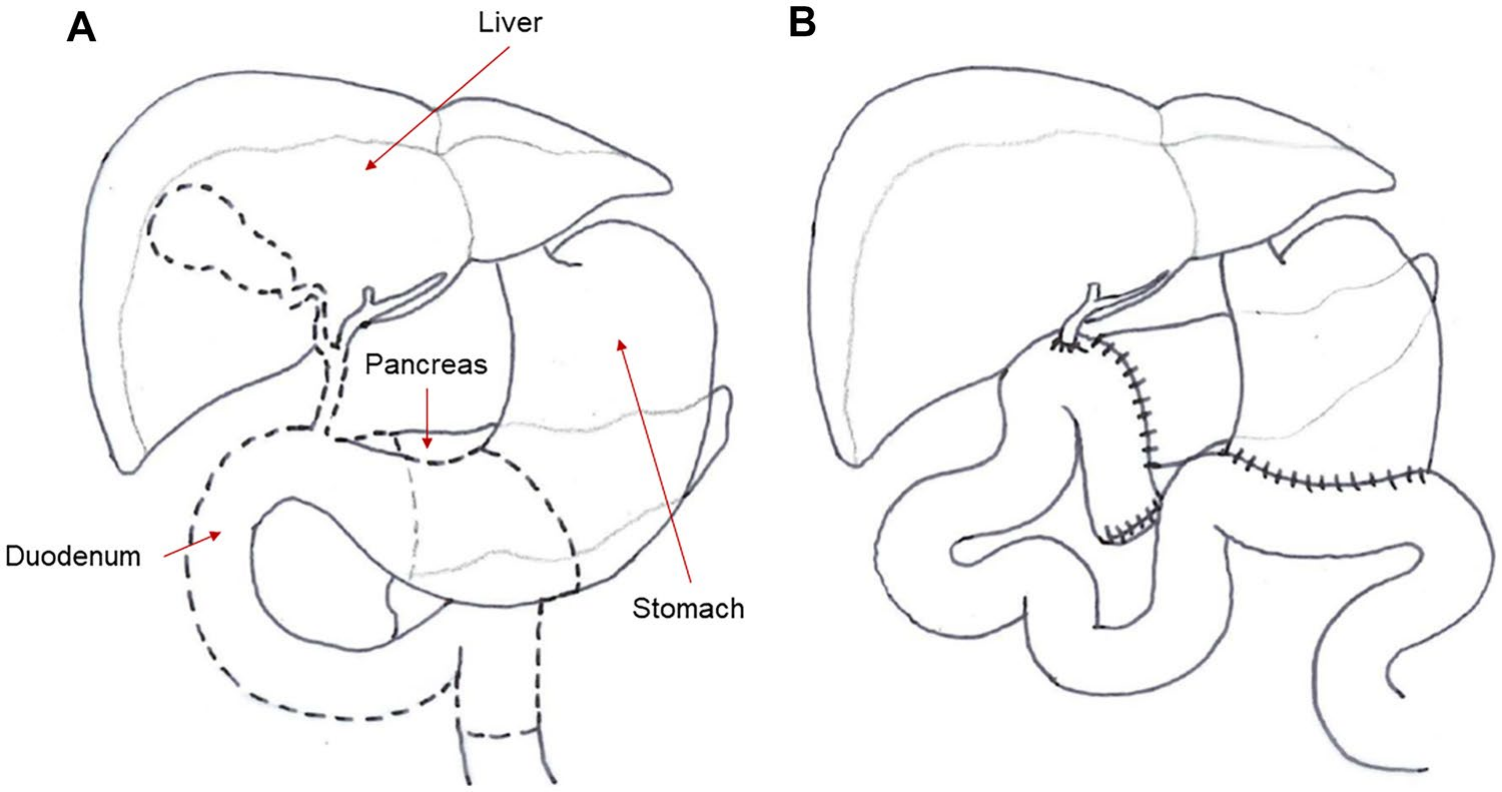
Pancreaticoduodenectomy (Whipple procedure). **A** Affected segments of the gastrointestinal tract (dashed lines) for a classic Whipple procedure. **B** Surgical reconstruction of the gastrointestinal tract in the classic Whipple procedure

Tumors of the small intestine are relatively rare. Both benign and malignant tumors can arise from the small intestine, with the main types being adenocarcinomas, sarcomas, neuroendocrine tumors, lymphomas, and gastrointestinal stromal tumors (GISTs) [39–41]. Tumors are generally treated with a combination of medical and surgical modalities [40]. With regard to surgical interventions [40, 41], endoscopic resection techniques are used for the treatment of localized tumors in the small intestine. Invasive lesions in the duodenum without major vessel involvement and distant spread are best treated by a pancreaticoduodenectomy (Whipple procedure) (Fig. 3). Segmental resection with regional lymphadenectomy is performed for tumors in the distal portions of the duodenum. Tumors in the jejunum and ileum are treated by wide excision, with the inclusion of areas of contiguous spread and the associated mesentery, with negative surgical margins.

Surgical resection is also performed for the treatment and staging of colorectal cancer. Segmental resections require adequate margins for clearance of any intramural spread, complete mesocolic excision, lymphadenectomy, and high ligation of the vascular supply [42, 43]. Surgical options for patients presenting with obstructing colon cancers include urgent resection with or without anastomosis, diversion, and endoscopic stenting with or without resection [44]. Anastomosis is dependent on the location of the tumor, the quality of the tissue, and the physiologic status of the patient [44]. Simple diversion and endoscopic stenting are reserved for patients that are unable to tolerate resection or with inoperable metastatic disease [44].

The type of surgical intervention for rectal cancer is dependent on the stage of the disease, as more invasive procedures can significantly affect patient morbidity [36, 45]. Local excision with transanal techniques involving negative circumferential and deep margins can be performed in early-stage disease to avoid the morbidity of proctectomy [45]. It should be noted that there is risk of luminal recurrence and occult metastatic disease with this procedure [45]. Radical excision is a major surgical procedure that involves abdominal dissection and removal of the primary tumor as well as the surrounding metastatic lymph node area. The main options include abdominoperineal resection (APR) and low anterior resection (LAR) [45–47]. In general, APR involves resection of the sigmoid colon, rectum, and anus—with the construction of an end colostomy [47]. LAR entails resection of the sigmoid colon and the rectum [46]. Intestinal continuity is restored for patients with tumors in the mid to distal rectum by forming an anastomosis to the descending colon. Diverting ileostomy or colostomy is commonly performed in patients undergoing proctectomy for rectal cancer [45].

## Physiological impact of gastric surgery on drug delivery

Surgical procedures on the stomach can alter gastrointestinal physiology, depending on the severity of the changes. These physiological changes can affect the effectiveness of orally administered formulations and should be considered in rational drug formulation design for specific pathological conditions that are commonly associated with surgical intervention (Table 1).

**Table 1:** Drug delivery considerations with gastric surgical procedures

**Physiological impact**	**Gastric surgical procedure**	**Drug delivery considerations**
**Dumping syndrome**	▪ Parietal cell vagotomy is associated with less frequency of the dumping syndrome in comparison to truncal vagotomy ▪ Resection of the pylorus can accelerate gastric emptying ▪ Dumping syndrome is a common complication of bariatric surgery (esp. RYGB and BPD)	▪ May affect drug formulations that are dependent on gastrointestinal transit time to trigger drug release, leading to reduce efficacy
**Increased gastric pH**	▪ Gastrectomy can lead to the production of less hydrochloric acid ▪ Surgical procedures that interfere with pyloric function may lead to reflux of alkaline duodenal fluid into the stomach	▪ May reduce the absorption of drugs that require an acidic environment for dissolution ▪ May trigger premature release of drugs from formulations that have pH-responsive coatings or matrices
**Post-surgery diarrhea**	▪ Truncal vagotomy may cause diarrhea (episodic or persistent) ▪ Bariatric surgery may cause diarrhea (e.g., diet high in fat can lead to diarrhea following BPD procedure)	▪ May reduce the time available for drug absorption ▪ May cause inefficient release of drugs from formulations that are dependent on gastrointestinal transit time ▪ Can affect other physiological factors in the gastrointestinal tract (e.g., intestinal volume, pH, mucosal integrity, and resident microbiome)
**Chronic gastroparesis**	▪ Gastroparesis can occur post-vagotomy when the vagus nerve is damaged	▪ May affect the time available for disintegration, dissolution, and/or drug absorption ▪ Premature drug release may occur in the stomach due to the prolonged gastric transit time
**Anastomoses**	▪ Gastric surgery with anastomoses is common with bariatric surgery (e.g., RYGB and BPD) ▪ Anastomoses may also be needed following other gastrectomy procedures to ensure alimentary continuity	▪ Until the anastomoses are healed, drugs should be delivered in their immediate-release formulation and ideally in a liquid dosage form or crushed ▪ After anastomoses are healed, whole pills can be administered (< 11 mm in size) ▪ Consider other routes of drug administration

### Dumping syndrome

Following a gastrectomy procedure, many patients will experience symptoms associated with the dumping syndrome to varying degrees depending on the type of surgical intervention [48–50]. This is due to the impairment of the stomach to regulate its rate of emptying. For example, parietal cell vagotomy is associated with less frequency of dumping syndrome in comparison to truncal vagotomy [51]. In addition, resection of the pylorus can accelerate gastric emptying [2, 49]. Dumping syndrome is also a common complication of bariatric surgery [49, 52]. Symptoms generally present shortly after eating and include gastrointestinal symptoms (e.g., nausea, vomiting, diarrhea, abdominal cramps, belching) and/or cardiovascular symptoms (e.g., weakness, flushing, sweating, palpitations, dyspnea). This response involves neural and hormonal mechanisms [48, 49]. These symptoms will usually resolve within a few months, but it can remain a clinical problem in a small percentage of patients. Dietary therapy that is low in carbohydrate and high in fat and protein content has been utilized in some indications to minimize symptoms by reducing jejunal osmolality [48, 49]. Drugs that slow gastrointestinal motility, such as anticholinergic drugs, may be beneficial following some surgical procedures [3]. Dumping syndrome is likely to adversely affect drug formulations that are dependent on gastrointestinal transit time to trigger drug release, leading to reduced efficacy in patients [3].

### Increased gastric pH

Gastrectomy, including gastric carcinoma resections or gastric bypass procedures (e.g., RYGB), can lead to the production of less hydrochloric acid [53]. In addition, surgical procedures that interfere with pyloric function (e.g., gastrojejunostomy) may lead to the reflux of alkaline duodenal fluid into the stomach [54]. This increase in gastric pH may reduce the absorption of drugs that require an acidic environment for dissolution (e.g., ketoconazole, atazanavir, rilpivirine) [55]. It can also trigger the premature release of drugs from formulations that have pH-responsive coatings or matrices [3]. These formulations are generally used for drugs that can irritate the gastric mucosa and for those that are susceptible to degradation by gastric enzymes or acidic pH [56–58].

### Post-surgery diarrhea

Diarrhea is a complication of some gastric surgery procedures. Truncal vagotomy may cause diarrhea, with the severity of the diarrhea varying between episodic symptoms and more persistent daily symptoms [11, 59]. These patients will likely require treatment with antidiarrheal agents. It should be noted that bariatric surgery can also cause diarrhea in patients [20, 21, 53]. For example, the malabsorption induced by the BPD procedure can be considerable and lead to debilitating diarrhea if a diet high in fat is consumed [53]. Rapid intestinal transit may result in less time for drug absorption as well as the inefficient release of drugs from formulations that are dependent on gastrointestinal transit time [3]. This can significantly affect the therapeutic efficacy of oral drug formulations. Diarrhea can also affect other physiological factors in the gastrointestinal tract, including the intestinal volume, pH, mucosal integrity, and resident microbiome [3].

### Chronic gastroparesis

Chronic delayed gastric emptying is occasionally seen after gastric surgery [60]. The vagus nerve controls the movement of food from the stomach through the gastrointestinal tract. Gastroparesis can occur post-vagotomy when the vagus nerve is damaged [11, 12]. Symptoms may improve with small and frequent feedings. Prokinetic agents (e.g., metoclopramide and domperidone) are often beneficial for these patients [3]. In refractory cases, total gastrectomy with esophagojejunostomy reconstruction may be required [11, 12]. Gastroparesis can have an impact on the effectiveness of orally administered drug formulations by affecting the time available for disintegration, dissolution, and/or drug absorption [3]. Premature drug release may occur in the stomach due to the prolonged gastric transit time.

### Anastomoses

Bariatric surgery poses a number of issues for the delivery of orally administered drugs during the immediate and long-term postoperative period [61–65]. Until the anastomoses are healed (~ 2 months postoperative), drugs should be delivered in a liquid formulation (if available) or crushed to allow adequate gastric delivery [61, 64]. Most sustained-release formulations should not be crushed and should instead be converted to their equivalent immediate-release formulation [61, 64]. Exceptions include those containing microencapsulated sustained release particles, whereby the capsule may be opened and the sustained released pellets dispersed in a small amount of water before administration. It is important for these pellets to remain intact and not be crushed. Other routes of administration should also be considered (e.g., intravenous and sublingual), especially with the occurrence of gastric leaks or failure of the anastomoses. After complete healing of the anastomoses, patients may be administered whole pills (e.g., tablets and capsules) in which the length and width are < 11 mm in size [61, 64]. Since gastrointestinal transit time is shortened with gastric bypass procedures, enteric-coated and sustained-release formulations should be avoided as they can result in erratic drug absorption. Instead, the use of immediate-release formulations are preferred [61, 64, 65].

## Physiological impact of bowel surgery on drug delivery

Bowel surgery can affect the gastrointestinal environment and, therefore, the effectiveness of orally and/or rectally administered formulations [3, 7]. The significance of the surgical intervention is dependent on the specific region of resection, the amount of bowel removed, the adaptation and absorptive capacity of the remaining bowel, and the nature of the underlying disease. This section will discuss the main physiological changes that should be considered for rational drug formulation design as well as when prescribing drugs for patients post-surgery (Table 2).

**Table 2:** Drug delivery considerations with general surgical bowel procedures

**Surgical issue**	**Physiological impact**	**Drug delivery considerations**
**Short bowel syndrome**	▪ Occurs following large resections of the small bowel (usually greater than 50%) ▪ May lead to significant impairment in the absorption of water, electrolytes, and nutrients ▪ Can alter luminal pH and transit times, reduce small chain fatty acid digestion, and impair regulation of the ileal brake ▪ Gastric hypersecretion may increase the acid load delivered to the duodenum	▪ May cause inefficient disintegration and drug release from formulations that are dependent on gastrointestinal transit time, leading to reduce efficacy ▪ Reduction in duodenal pH may delay drug release from formulations that have pH-responsive coatings or matrices that are designed to release drug into the small intestine for optimal absorption
**Resection of the terminal ileum**	▪ May reduce the ileal brake and cause rapid intestinal transit ▪ Can cause choleretic diarrhea, due to the terminal ileum being responsible for bile salt reabsorption	▪ May reduce the time available for drug absorption from both oral and rectal dosage forms ▪ May cause inefficient release of drugs from formulations that are dependent on gastrointestinal transit time ▪ Diarrhea can affect other physiological factors in the gastrointestinal tract (e.g., intestinal volume, pH, mucosal integrity, and resident microbiome)
**Resection of the colon**	▪ Colonic resections may alter the local microenvironment and physiology of the gastrointestinal tract	▪ May cause inefficient release of drugs from formulations that are dependent on gastrointestinal transit time ▪ May affect pH-dependent dosage forms that utilize the drop in pH on entry into the colon ▪ May affect formulations that exploit the metabolic capabilities of the colonic microbiome
**Surgical intervention for rectal cancer**	▪ May cause functional complications that increase intestinal motility and transit (e.g., urgency and frequent bowel movements)	▪ May reduce the time available for drug absorption ▪ May cause inefficient release of drugs from formulations that are dependent on gastrointestinal transit time ▪ Diarrhea can affect other physiological factors in the gastrointestinal tract (e.g., intestinal volume, pH, mucosal integrity, and resident microbiome)
**Intestinal stoma**	▪ An ileostomy produces a relatively continuous stream of liquid and semi-solid material ▪ Can significantly change the absorption of water and electrolyte in the gastrointestinal tract, leading to a relatively high output ▪ Patients are at risk for developing electrolyte depletion (e.g., hyponatremia) and dehydration ▪ Colostomy tends to produce more formed stool and less risk of metabolic disturbances	▪ Can affect the release of drugs from formulations that are dependent on pH and transit time (e.g., enteric-coated and sustained-release formulations) ▪ Use of immediate-release formulations are preferred to improve drug absorption in patients, especially those with an ileostomy

### Short bowel syndrome

Short bowel syndrome generally occurs following large resections of the small bowel (greater than 50%), thereby leaving a residual small intestine of approximately 2 meters or less [66–68]. This can lead to significant impairment in the absorption of water, electrolytes, and nutrients (carbohydrates, protein, fat, and vitamins) [66, 69]. Although some adaptive changes are seen in the remaining intestine, large resections may cause functional and physiological changes such as altering luminal pH and transit times, reducing small chain fatty acid digestion, and impairing regulation of the ileal brake (mechanism that slows transit times for nutrient absorption) [68, 70, 71]. For example, short bowel syndrome may cause gastric hypersecretion and increase the acid load delivered to the duodenum. The reduction in duodenal pH can inhibit the function of digestive enzymes and further impair the absorption of nutrients [66, 68, 69].

With regard to drug delivery, shortening of the intestine can potentially affect the way oral formulations are processed [2, 68, 69]. The reduction in duodenal pH may delay drug release from formulations that have pH-responsive coatings or matrices that are designed to release the drug into the small intestine for optimal absorption [56–58]. In addition, formulations that are dependent on gastrointestinal transit times to activate drug release may also be affected, causing inefficient disintegration and drug release as well as reduced efficacy [3]. These formulations typically rely on the relatively constant transit time through the small intestine, which will be adversely affected in short bowel syndrome.

### Resection of specific intestinal regions

Resections of specific regions of the bowel can affect drug delivery. For example, removal of the terminal ileum affects water absorption, thereby diluting residual bile acids in the colon and reducing net colonic fatty acid concentrations [72, 73]. The decrease in fatty acids reduces the ileal brake, causing more rapid intestinal transit [70, 71]. Removal of the terminal ileum can also cause choleretic diarrhea, due to this region being responsible for bile salt reabsorption [2]. The increase in intestinal transit can significantly affect the time available for drug absorption from both oral and rectal dosage forms, as well as the efficacy of oral formulations that depend on gastrointestinal transit time to trigger drug release [3]. Diarrhea can also affect other physiological factors in the gastrointestinal tract, including the intestinal volume, pH, mucosal integrity, and resident microbiome [3].

In addition, surgical intervention for rectal cancer may also lead to functional complications that impact the effectiveness of conventional oral formulations [36, 45]. This includes the low anterior resection syndrome (LAR syndrome) which is due to a combination of factors such as colonic dysmotility, nerve injury, and aberrant anal canal sensation [74, 75]. Symptoms include urgency and frequent bowel movements, thereby increasing intestinal motility and transit [74, 75]. Surgical resection of the colon can also alter the local microenvironment and physiology of the gastrointestinal tract [2, 3]. This may affect the efficacy of delayed-release dosage forms that rely on gastrointestinal transit time, pH-dependent dosage forms that utilize the drop in pH on entry into the colon, and formulations that exploit the metabolic capabilities of the colonic microbiome [3, 76, 77].

### Intestinal stomas

Intestinal stomas are constructed by fixing a part of the bowel wall onto the surface of the abdomen. The two most common intestinal stomas are those made from the ileum (ileostomy) and colon (colostomy) [78]. An end stoma is created by fixing the end of the ileum or colon to the abdominal wall, whereas a loop stoma involves bringing a segment of the ileum or colon to the abdominal wall and opening the side of the bowel [78, 79]. The latter procedure creates a proximal and distal opening that allows retrograde decompression of the distal bowel and has the advantage of being easier to reverse [79]. Stomas are generally created to protect a distal anastomosis temporarily or as a permanent route for enteric contents when the colon and/or rectum is resected [78, 79].

An ileostomy produces a relatively continuous stream of liquid and semi-solid material [80]. It significantly changes the absorption of water and electrolytes in the gastrointestinal tract, due to the absence of the colon. Although the small intestine can somewhat adapt to this change, output can still remain relatively high compared to normal [80, 81]. Therefore, patients are at risk for developing electrolyte depletion (e.g., hyponatremia) and dehydration. Output can be higher in patients with diseased small bowel or more proximal stomas [80, 81]. Dietary modifications, fiber supplements, and antimotility agents can be added as needed to manage high output [80, 81]. In terms of drug delivery considerations, the physiological impact of a stoma can affect the release of drugs from formulations that are dependent on pH and transit time (e.g., enteric-coated and sustained-release formulations) [3]. Therefore, the use of immediate-release formulations are preferred to improve drug absorption in patients, especially those with an ileostomy [82]. Colostomy tends to produce more formed stool and patients have a reduced risk of developing metabolic disturbances [80].

## Pharmaceutical considerations for conventional single-unit drug delivery approaches

Gastric and bowel surgery may significantly alter the functionality of the gastrointestinal tract, thereby affecting the way the body processes oral formulations. The main dosage forms used for gastrointestinal drug delivery are solid dosage forms (e.g., tablets and capsules) and liquid dosage forms (e.g., solutions and suspensions) [83]. Conventional formulations can be modified to control the location in which the drug is released in the gastrointestinal tract for subsequent absorption—namely the stomach, the small intestine, and the large intestine. The pharmaceutical considerations influencing conventional drug delivery approaches in patients that have had gastrointestinal surgery will be discussed.

### Impact of gastrointestinal surgery on conventional gastroretentive dosage forms

Gastroretentive formulations prolong the gastric residence time of drugs to provide sustained or controlled release in the stomach and/or small intestine for absorption [84–87]. Although the majority of these dosage forms are still in the research and development stage, it is worth noting the advantages and limitations of such formulations for patients that have undergone general surgical procedures on the gastrointestinal tract. Dosage forms that are designed to float over gastric content are the most common commercialized gastroretentive drug delivery system [88]; however, they require sufficient stomach content to allow an effective separation between the dosage form and the pylorus [89, 90]. This can be affected by gastric resections and restrictions, which can significantly reduce the volume of the stomach. Expandable dosage forms that enlarge or swell in the stomach to dimensions that prevent premature emptying through the pylorus have also been developed [89, 91]. Surgical procedures that result in a decrease or increase in the size of the gastroesophageal or gastroduodenal junction can potentially lead to prolonged retention and accumulation of several dosage units in the stomach or premature loss of the dosage form, respectively. Mucoadhesive dosage forms to the gastric mucosa and high-density dosage forms that sink into the folds of the antrum are less common approaches [3]. Understandably, mucoadhesive dosage forms can suffer from unpredictability regarding the site of adhesion [91, 92], and high-density dosage forms can be impacted by surgical removal of the distal portion of the stomach (e.g., antrectomy) [87, 93]. In terms of possible advantages of gastroretentive dosage forms in patients post-surgery, they may be beneficial to increase drug concentrations in the stomach to treat local conditions (e.g., infections) or allow prolonged drug release into the small intestine to optimize systemic drug absorption. The ideal formulation is highly dependent on the type of gastric surgery and the subsequent degree of alteration in gastrointestinal physiology.

### Impact of gastrointestinal surgery on conventional dosage forms targeting the small intestine

The main conventional formulations that target the small intestine are pH-dependent dosage forms and mucoadhesive dosage forms. pH-dependent dosage forms are comprised of pH-responsive coatings or matrices that are typically composed of polymers that are soluble at specific pH ranges, such as hydroxypropyl methylcellulose (HPMC) derivatives [94, 95] and some methacrylic resins (commercially available as Eudragit^®^) [56, 96, 97]. In particular, enteric-coated solid dosage forms (e.g., capsules and tablets) are commonly used clinically [56, 98]. Advantages of such formulations include the protection of drugs that are susceptible to causing irritation to the gastric mucosa or degrading in the stomach [56–58], as well as creating an extended or delayed drug release profile for controlled drug delivery [76, 99]. It is important to note that significant intra- and inter-individual variability in the pH of the gastrointestinal tract can occur in healthy individuals [100–106]. General surgical procedures can further alter the pH of the gastrointestinal tract, thereby affecting the performance of pH-dependent dosage forms. For example, gastric resections that involve surgical removal of a significant portion of the gastrin-producing mucosa (e.g., antrectomy or subtotal gastrectomy) or surgical procedures that interfere with pyloric function (e.g., gastrojejunostomy) can increase the pH in the stomach and trigger the premature release of drugs from pH-dependent formulations. Similarly, short bowel syndrome may cause gastric hypersecretion, thereby increasing the acid load delivered to the duodenum and reducing the pH in this region. In addition, surgical interventions that increase gastrointestinal transit (e.g., bowel surgery) may also cause erratic drug release from pH-dependent formulations by not allowing sufficient time for the dissolution or degradation of the polymers [98, 107].

Mucoadhesive dosage forms (e.g., intestinal patches) have been developed as a means to prolong contact with the intestinal mucosa to improve drug absorption and to provide protection to drugs that are susceptible to degradation in the upper gastrointestinal tract [108–112]. It is unlikely that general surgical procedures on the gastrointestinal tract will significantly affect the performance of such dosage forms, unless significant portions of the small intestine have been resected. Inherently, mucoadhesive dosage forms require sufficient binding with the intestinal wall to be effective [4, 109]. However, there is a risk of premature adhesion to other mucosal surfaces in the upper gastrointestinal tract following oral administration or adhesion to different segments of the small intestine. Similar to gastroretentive dosage forms, advantages of the mucoadhesive dosage form in patients post-surgery include the ability to increase drug concentrations in the small intestine to treat local conditions (e.g., infections) or allow prolonged drug release into the small intestine to optimize systemic drug absorption.

### Impact of gastrointestinal surgery on conventional dosage forms targeting the large intestine

Conventional formulations that target the large intestine are particularly useful for the treatment of local diseases affecting the colon (e.g., colorectal cancer and IBD) to enhance the local efficacy of therapeutics as well as to reduce the risk of systemic side effects. One of the common approaches is the use of pH-dependent dosage forms to release drugs into the distal part of the small intestine or colon [76, 99]. For example, mesalazine is clinically available as tablets coated with Eudragit S (Asacol^®^) or Eudragit L-100 (Mesasal^®^ and Colitofalk^®^) for use in IBD. The potential issues surrounding pH-dependent formulations in patients that have undergone gastrointestinal surgery have been addressed above (refer to “Impact of gastrointestinal surgery on conventional dosage forms targeting the small intestine”).

Time-dependent dosage forms, which rely on time-dependent mechanisms to trigger drug release based on the transit times through various regions of the gastrointestinal tract, have also been investigated for targeting the colon. In general, these dosage forms contain hydrophilic polymers in the matrix or coating (e.g., HPMC and ethyl cellulose) that swell gradually over time, thereby establishing a lag phase before triggering drug release [95, 113, 114]. The drug release rate is controlled by the gel layer that is formed when the polymer is hydrated following contact with aqueous fluids [115]. Formulation factors can be modified to control drug release. This includes altering the polymer composition, distribution, concentration, and viscosity as well as drug solubility, loading, and particle size [115–117]. These formulations assume that the transit time through the stomach and small intestine is relatively constant. However, it is well established that gastrointestinal transit time can vary significantly in the stomach, small intestine, and colon based on physiological factors [6, 31, 101, 118]. With regard to general surgical procedures, transit times can be altered temporarily or permanently. An increase in gastrointestinal transit (e.g., short bowel syndrome, dumping syndrome, post-surgery diarrhea, gastric bypass procedures, resection of the terminal ileum, surgical intervention for rectal cancer, and intestinal stomas) may lead to inadequate time for drug release from these formulations [3]. Conversely, a decrease in gastrointestinal transit (e.g., chronic gastroparesis post-gastric surgery) may lead to the release of the drug into an earlier segment of the gastrointestinal tract, which can affect the efficiency of drug absorption and the effectiveness of the drug [3].

Biodegradable dosage forms are another key approach for targeting the large intestine. These formulations exploit the resident colonic bacterial flora, which produce numerous enzymes (e.g., azoreductases and polysaccharidases) that are able to breakdown biodegradable polymers in coatings and/or matrix formulations to trigger drug release into the large intestine [119–121]. For example, azoreductase activity of colonic bacteria has been exploited in the design of prodrugs of 5-aminosalicylic acid (5-ASA), such as olsalazine and sulfasalazine, which are used in the treatment of IBD [122]. Similarly, non-starch polysaccharide formulations are commonly used for biodegradable dosage forms, as they are more resistant to digestion and absorption in the small intestine compared to the large intestine [120, 123, 124]. It should be noted that these polymers, which are typically hydrophilic, are able to hydrate and swell during gastrointestinal transit. Colonic bacteria and enzymes then penetrate the hydrated layers and degrade the polymer to trigger the release of drugs [76, 124]. Premature drug release can, therefore, occur with prolongation in gastrointestinal transit time (e.g., chronic gastroparesis post-gastric surgery) [6, 89, 105, 125–128]. Furthermore, inefficient degradation can occur due to dysbiosis when the colonic microbiome is disrupted (e.g., post-surgery diarrhea and use of antibiotics for post-surgery infections) or from an increase in gastrointestinal transit (e.g., short bowel syndrome, dumping syndrome, post-surgery diarrhea, gastric bypass procedures, resection of the terminal ileum, surgical intervention for rectal cancer, and intestinal stomas) [3, 129, 130].

## Pharmaceutical considerations for multiparticulate drug delivery approaches

Multiparticulate formulations have gained increasing interest for gastrointestinal drug delivery compared to conventional single-unit dosage forms. In these formulations, the dose of the drug is distributed across a number of individual subunits rather than in one single unit. Single-unit dosage forms encounter issues such as dose dumping, unpredictable disintegration and dissolution, and stability issues in the gastrointestinal tract [77, 131, 132]. This is overcome with the use of multiparticulate formulations that generally have a larger surface-area-to-volume ratio, thereby providing an increased surface area for the solubilization of drugs and interaction with the mucosal surface. For example, Entocort^®^ EC is a commercially available multiparticulate formulation of budesonide (corticosteroid) for the treatment of colonic inflammation, particularly for IBD [133, 134]. The dosage form contains ethyl cellulose–based granules that are approximately 1 mm in size, with each granule coated with Eudragit^®^ L (pH-responsive coating) to allow drug release in the ileum and ascending colon.

The development of nanoparticulate dosage forms has also gained increasing interest, as the smaller size of these subunits has been shown to confer several unique advantages for gastrointestinal drug delivery. Nanoparticulate formulations for drug delivery essentially are drug-containing nanoparticles that aim to increase therapeutic efficacy, decrease the therapeutically effective dose, and/or reduce the risk of systemic side effects [135]. Advantages of nanoparticulate formulations for oral drug delivery include more uniform distribution and drug release, easier transport through the gastrointestinal tract, improved interaction and uptake into mucosal tissues and cells, increased retention of the nanoparticles in the gastrointestinal tract (even during diarrhea), and specific accumulation to the site of disease (e.g., inflamed tissues) [77, 136, 137]. These innovative platforms have been investigated for regional targeting to the stomach, small intestine, and large intestine [3]. For example, prolonged gastroretention of up to 3 h was demonstrated for nanoparticulate dosage forms in animals that had been fasted in initial in vivo biodistribution studies [138]. Similarly, microparticulate dosage forms were shown to prolong gastric retention to over 8 h in the fasted state [139]. In addition, nanoparticulate formulations have been shown to enhance colonic residence time and mucosal uptake in inflamed intestinal regions by exerting an epithelial enhanced permeability and retention (eEPR) effect [140, 141]. Consequently, they are able to avoid rapid carrier elimination by diarrhea, which is a common symptom in IBD [142]. Conventional formulations do not have this advantage as they are generally designed to promote regional deposition of the drug in the gastrointestinal tract. Nanoparticulate formulations can be modified to deliver drugs via various mechanisms such as passive targeting, active targeting, solubilization, and triggered release [135].

Multiparticulate formulations, especially using nanomedicine technology, are a promising and innovative approach for gastrointestinal drug delivery. However, there is currently very limited data on the pharmacokinetics and pharmacodynamics of nanomedicines after gastrointestinal surgery in the available literature. For example, Chen et al. [143] were the first to investigate the usefulness of nanoparticulate formulations for improving drug malabsorption elicited by gastric bypass surgery. In particular, they evaluated the efficacy of self-nanoemulsifying drug delivery systems (SNEDDS) for enhancing the oral delivery of the poorly water-soluble compound, silymarin, in rats that had undergone RYGB surgery. The nanoparticles (~ 190 nm in diameter) enhanced the oral bioavailability of the compound in RYGB rats by 2.5-fold and 1.5-fold compared to the free drug suspension and PEG 400 solution, respectively. Further studies are needed to comprehensively explore the use of nanomedicines for oral drug delivery following various general surgical procedures on the gastrointestinal tract. The data will inform the translational development of this novel drug delivery platform to improving gastrointestinal drug delivery for this specific patient population.

It should be noted that gastrointestinal surgeries are common in the clinic for patients with a range of underlying conditions (e.g., IBD, gastrointestinal cancers, bowel obstruction, and bariatric surgery). These procedures generally cause permanent changes to the anatomy and/or physiology of the gastrointestinal tract and can limit the types of conventional pharmaceutical formulations that can be administered. Innovative drug delivery systems are warranted for these patients, as the post-surgical changes to the gastrointestinal tract may lead to malabsorption of orally administered drugs. Patients may also benefit from targeting and accumulation of drugs in specific regions of the gastrointestinal tract to treat local conditions post-surgery (e.g., inflammation or infection) as well as to improve their compliance to regular medications with formulations that require less frequency in dosing.

## Conclusion

General surgical interventions on the stomach and bowel can significantly alter the anatomy and physiology of the gastrointestinal tract, thereby affecting gastrointestinal drug delivery. These factors should be considered when prescribing drugs, including dosage form and dose, for patients. Consideration of these factors in the development of novel formulations for specific indications that commonly require gastrointestinal surgery will enable optimized drug delivery.

## Data Availability

Not applicable.
